# Opportunities to Improve Animal Welfare during Transport and Slaughter of Cattle and Pigs through Staff Training—Results of a Delphi Survey

**DOI:** 10.3390/ani13243859

**Published:** 2023-12-15

**Authors:** Fabienne Eichler, Veronica Duckwitz, Rudi Isbrandt, Svea Nicolaisen, Nina Langkabel, Mechthild Wiegard, Diana Meemken, Christa Thöne-Reineke, Marcus G. Doherr

**Affiliations:** 1 Institute of Veterinary Epidemiology and Biostatistics, School of Veterinary Medicine, Freie Universität Berlin, 14163 Berlin, Germany; 2 Institute of Food Safety and Food Hygiene, School of Veterinary Medicine, Freie Universität Berlin, 14163 Berlin, Germany; 3 Institute of Animal Welfare, Animal Behavior and Laboratory Animal Science, School of Veterinary Medicine, Freie Universität Berlin, 14163 Berlin, Germany

**Keywords:** animal welfare officer, bovine, expert elicitation, online training, porcine

## Abstract

**Simple Summary:**

Staff of transport companies and abattoirs who handle live cattle and pigs have a high degree of responsibility for animal welfare. However, there are frequent reports of animal welfare concerns regarding the handling of cattle and pigs during transport and slaughter. Continuous training on the correct and proper handling of animals is suitable for strengthening animal welfare. For that purpose, online training modules are developed at the Freie Universität Berlin, Germany. Experts in this field were asked to rate predetermined action points according to their relevance for animal welfare during transport and slaughter, and their potential for improvement through targeted training in two consecutive survey rounds (=Delphi approach). The rating scale included scores from 0 (=‘not relevant’, respectively ‘no possibility of improvement’) to 10 (=‘very relevant’, respectively ‘very high possibility of improvement’). The experts rated ‘Assessment of fitness for transport’, ‘Unloading at abattoir’, ‘Handling at stunning’ and ‘Exsanguination’ as the most relevant action points. None of the action points were rated with a median score below 5. The Delphi approach was seen as a valuable method to include external expertise to select the most relevant action points for the online training modules.

**Abstract:**

To improve animal welfare for cattle and pigs during transport and at slaughter, online training modules for all staff including employees in the lairage pen, the slaughter line as well as animal welfare officers are developed at Freie Universität Berlin, Germany. Before starting the development of these modules, an expert elicitation survey using a modified Delphi approach was performed to identify action points considered most relevant for animal welfare during transport and slaughter, and as having the potential for improvement through training. In total, 49 participating experts rated predetermined action points of each step in the transport and slaughter process in two survey rounds. The rating scale included numbers 0 (=‘not relevant’, respectively ‘no possibility of improvement’) to 10 (=‘very relevant’, respectively ‘very high possibility of improvement’). None of the action points were rated with a median score of less than 5. Assessment of fitness for transport, unloading at the abattoir, handling at stunning and exsanguination were amongst the highest rated action points, and were therefore selected to develop online training modules. The Delphi approach was seen as a valuable method to include external expertise to select the most relevant action points for the development of online training modules.

## 1. Introduction

Animal welfare of cattle and pigs during transport to the abattoir and the slaughter process is an important issue worldwide [1,2]. In the European Union (EU), abattoir staff involved in slaughter operations must provide a certificate of competence but further training is not mandatory [3]. The animal welfare regulations that must be officially followed during transport and slaughtering are governed by legislation such as the Regulation (EC) No 1/2005 on the protection of animals during transport and related operations [4] and the Regulation (EC) No 1099/2009 on the protection of animals at the time of killing [3]. In Germany, additional legislation includes the Animal Protection Law (§ 4a Tierschutzgesetz) [5], and legislation which specify the Regulations of the EU such as the Animal Protection Transport Ordinance [6], the Animal Welfare Slaughter Ordinance [7] and the Federal Law on prohibitions of the trade of certain animal products and on the delivery of animals in certain cases (§ 4 Tiererzeugnisse-Handels-Verbotsgesetz). These regulations lay down the requirements for handling, immobilisation and stunning of animals to ensure animal welfare.

To ensure a high level of animal welfare, staff of transport companies and abattoirs who handle live animals require appropriate education and training to guarantee that each action is carried out according to the latest animal welfare standards. Training of staff has been noted as an important preventive measure for animal welfare hazards during transport and slaughter [8,9,10,11]. The main challenges are (i) finding the necessary time for the training of all staff and (ii) overcoming the substantial differences in language skills as well as cultural and educational backgrounds. Due to the diversity within the staff, animal welfare training varies greatly between individual abattoirs in Germany and Austria [12].

To address the educational requirements, the joint research project ‘Development of target group-specific e-learning modules to improve animal welfare during the transport and slaughter of cattle and pigs (eSchulTS^2^)’ was launched at Freie Universität Berlin as a collaborative project between various university institutes and an abattoir company as an industrial partner. The objective comprises the development of online training modules for transport and abattoir staff, including animal welfare officers involved in transportation and slaughter of cattle and pigs. Particular attention is paid to differences in language skills and educational backgrounds. The training modules are didactically adjusted according to the most commonly spoken languages of the respective staff at abattoirs in Germany and Austria: German, Romanian, Polish, Hungarian, Bulgarian [12] and English. They are made available to all stakeholders through a web-based platform free of charge.

Online training modules on animal welfare at slaughter are already used in the education of veterinary students [13,14]. The European Food Safety Authority (EFSA) has developed guides for good practices for the transport of animals, but an online course with a guided course structure for staff of transport companies or abattoir staff has not yet been described in the scientific literature. Online training modules allow participants to study at their own pace and without the need to be in a specific location at a specific time. This can be beneficial for transportation and abattoir staff with varying educational levels and different work schedules.

To identify important action points concerning animal welfare during transport and slaughter, appropriate systematic literature reviews were performed in advance for cattle and pigs [15,16]. To ascertain the most effective training subjects from the action points elucidated from the literature searches, a survey-based Delphi approach was deemed most appropriate. The aim of this survey was to assess the action points regarding their relevance for animal welfare and their potential for improvement of animal welfare through staff training.

## 2. Materials and Methods

### 2.1. General Aspects of the Delphi Approach

The Delphi approach describes a multi-stage qualitative survey tool which is used to achieve a reliable and evidence-based ranking of topics [17]. A group of selected experts is asked to form opinions concerning an undecided issue. First, the issue is divided into items. The experts are asked to rate specific questions concerning the items on a predefined scale. This rating is performed via a written survey. This way, each assessment is evaluated equally and there is no risk of individual experts not being heard or some experts dominating the opinion-forming process. When the answers are returned, median scores are calculated on each item and sent back to the experts for a second assessment. Here, experts compare their individual answers given in the first round with the median result of the whole expert group. In case of major deviations, they may reconsider and change their individual results or maintain their previous assessment. In both cases, an explanation is asked for. This can be followed by further rounds of surveying or group discussions depending on the aimed level of consensus between all experts [17].

### 2.2. Compiling the Delphi Survey

Based on systematic literature reviews, the topics of transport and slaughter of cattle and pigs were each subdivided into five process steps (Table 1). To further specify the process steps between farm of origin and abattoir, two to six action points with characterising examples were defined for each process step. Each topic area was divided into 16 to 19 action points (see Supplementary File Table S1). Each action point was to be rated by the experts regarding its relevance to animal welfare and potential improvement through online training. Specific questions for a rating assessment on a scale of 0 to 10 were compiled:

1. How relevant is the named action point for animal welfare aspects?
  - Not relevant at all (0) to highly relevant (10)
2. To what extent can a targeted training of staff involved in this action point improve animal welfare?
  - No potential of improvement at all (0) to very-high potential of improvement (10)
3. To what extent can a targeted training of animal welfare officers in this action point improve animal welfare?
  - No potential of improvement at all (0) to very-high potential of improvement (10)

### 2.3. Selection of the Group of Experts 

The project team contacted individuals working in the field of animal welfare, meat hygiene, Veterinary Public Health (VPH) and in meat industry facilities to compile a list of possible experts in transport and slaughter of cattle and pigs. The experts were divided into three categories: experts based in research institutions, experts based in the industry and experts based in meat inspection and animal welfare (Table 2). Overall, 104 experts were contacted via e-mail to participate in the Delphi survey. As the Delphi approach requires that experts receive personalised surveys to be able reassess their first rating in a second round, they were informed accordingly in the first invitation and by responding consented that the elicitation would not be anonymous, but that responses would be kept confidential and not shared with other experts. They were also informed about the purpose of the survey and that their assessments would aid in the preparation of publicly available online training modules. 

### 2.4. Assessment Process 

**Figure 1:**
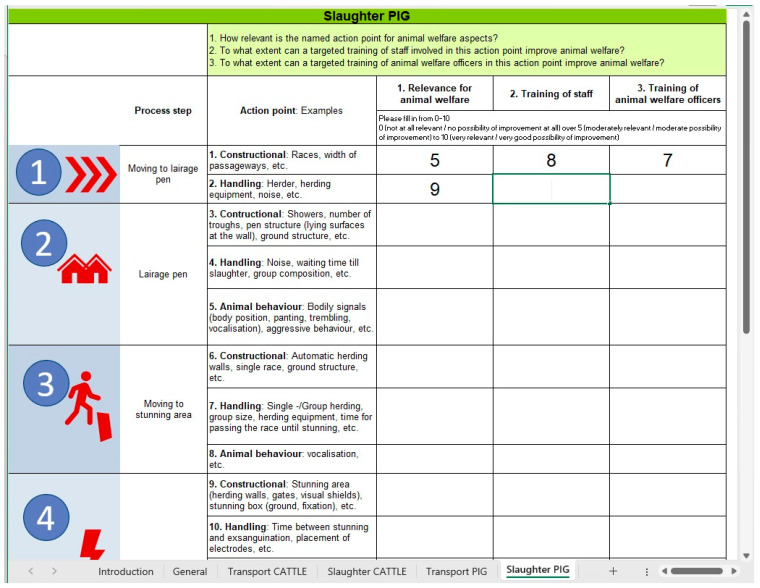
Screenshot of MS Excel^©^-based workbook (Supplementary File Table S1) used for the Delphi survey on animal welfare relevance and training potential of processing steps along the transport and slaughter chain for cattle and pigs (Table 1). Spreadsheet ‘Slaughter PIG’ is partly filled out with numbers between 0 and 10 as an example.

The Delphi survey took place in two consecutive rounds in May and June 2021. An MS Excel^©^-based workbook with six spreadsheets (one introductory sheet, one sheet for demographic data and four sheets for the respective topic areas) was used as a data collection tool (Figure 1, Supplementary File, Table S1). The spreadsheets were pre-tested by doctoral students at the Institute of Veterinary Epidemiology and Biostatistics, School of Veterinary Medicine of Freie Universität Berlin, Germany, who were not involved in the project. 

In the first round, the experts were given two weeks to rate all action points regarding the three questions mentioned above. Exceptions were made for the first four process steps (‘Route planning/time management’ to ‘Transportation’) in the topic areas ‘Transport CATTLE’ and ‘Transport PIG’. These process steps were only to be rated on questions number 1 and 2 (‘Relevance for animal welfare’ and ‘Possible improvement through staff training’), as animal welfare officers are not involved there. In total, the experts were asked to rate 183 items (two to three relevant questions for each action point; see Figure 1, Supplementary File, Table S1). A reminder was sent four days before the deadline. The returned spreadsheets were evaluated and the median scores of all responses were derived for each action point. The median scores were then added as a new column into the scoring sheets. 

In the second round, all experts contributing to the first round received the new MS Excel^©^-based workbook with the respective experts’ answers from the first round and the median score value over all experts. They were asked to compare their individual scores from the first round with the median score. If the score differed significantly (defined as ≤3/≥3 from median score values), they were specifically asked to consider whether they would like to adjust their assessment and comment on their decision (i.e., change their first assessment or stay with it). Again, the experts were given two weeks to complete this second assessment. After ten days, a short reminder was sent to the experts who had not yet returned their answers.

### 2.5. Statistical Analyses

The median, arithmetic mean, standard deviation (SD), coefficient of variation (CV), minimum and maximum of scores given for each topic area and both rounds were calculated in MS Excel^©^ version 2016. For better readability, the median over all experts (rounded to full numbers) was reported back to the experts in the second round. For graphical visualisation, the results for all action points were ranked by the mean scores and presented in scatter plots. Action points ranking high on both scales (relevance and training potential; upper right quadrant in the plots) were given priority for the development of the training platform.

For further statistical analysis, the R package ‘ggridges’ (R Core Team 2021, version 4.1.2.) was used for the illustration of ridgeline plots [18]. For comparison of the three subgroups, an overall Kruskal–Wallis rank-sum test with the means of the subgroups was performed for the dependent variable and the different subgroups for the independent variable.

## 3. Results

### 3.1. Response Rate

**Figure 2:**
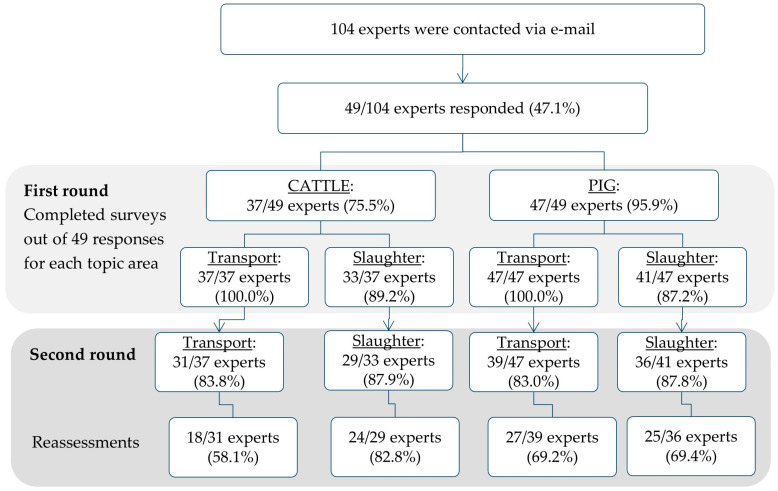
Flowchart depicting both rounds of the Delphi survey on animal welfare relevance and training potential of processing steps along the transport and slaughter chain for cattle and pigs. Response rates are presented for each topic area. The percentages refer to the respective number above.

In the first survey round, 49 experts returned their spreadsheets (response rate: 47.1%, 49/104). Of these 49 experts, a total of 37 filled out the spreadsheet for ‘Transport CATTLE’ and 33 for ‘Slaughter CATTLE’. The spreadsheets for ‘Transport PIG’ and ‘Slaughter PIG’ were completed by 47 and 41 experts, respectively (Figure 2).

Of the 49 experts from the first round, 41 responded again in the second round (response rate: 83.7%, 41/49). Overall, 31 out of the 41 experts filled out the spreadsheet for ‘Transport CATTLE’ and 29 of them for ‘Slaughter CATTLE’. The spreadsheets for ‘Transport PIG’ and ‘Slaughter PIG’ were filled out by 39 and 36 experts, respectively. Reassessments were performed by 58.1% (18/31) of the experts in the topic area ‘Transport CATTLE’. A total of 82.8% (24/29) of the experts did so for the topic area ‘Slaughter CATTLE’. Similarly, 69.2% (27/39) of the experts made at least one reassessment in the topic area ‘Transport PIG’ and 69.4% (25/36) of the experts for ‘Slaughter PIG’ (Figure 2).

All experts experienced in meat inspection and animal welfare who participated in the first round also responded in the second round (10/10). Most non-responders in absolute numbers from first to second round were found in the group of experts employed in research institutions (5/26), followed by the meat industry experts (3/13). There were no significant differences between the answers of the three subgroups. In the second round, some experts have chosen to comment on their decision on a specific item’s rating (maintaining or reassessing the original score). One exemplary comment on a specific item from each topic area can be found in Table 3.

### 3.2. Scoring

**Figure 3:**
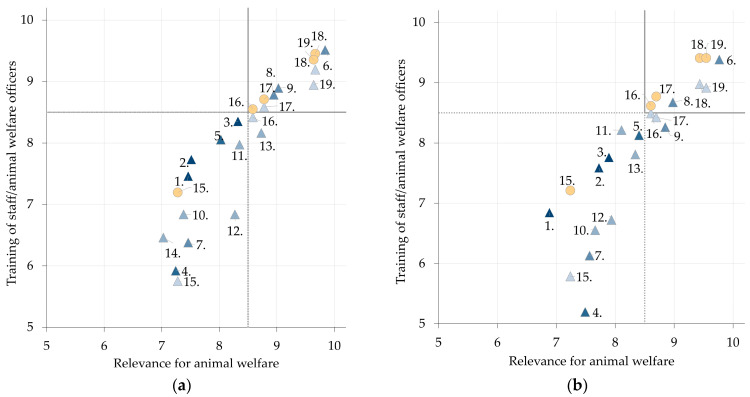
Scatterplots showing the average scores for all experts on action points in the topic areas ‘Transport CATTLE’ (**a**) and ‘Transport PIG’ (**b**) scored in the second round of the Delphi survey on animal welfare relevance and training potential of processing steps along the transport and slaughter chain for cattle and pigs. Blue triangles represent the relationship between rating of action points on ‘Relevance for animal welfare’ (*X*-axis) and ‘Training of staff’ (*Y*-axis). Orange circles represent the relationship between rating of action points on ‘Relevance for animal welfare’ (*X*-axis) and ‘Training of animal welfare officers’ (*Y*-axis). Different shadings of triangles refer to the categorisation of the action point of the respective process step (dark to bright = process steps 1 to 5). Numbers relate to individual action points (see Supplementary File Table S1). High scores (>8.5) relate to a high relevance for animal welfare aspects and a high possibility to improve animal welfare through training. Further details can be found in Figure A1.

**Figure 4:**
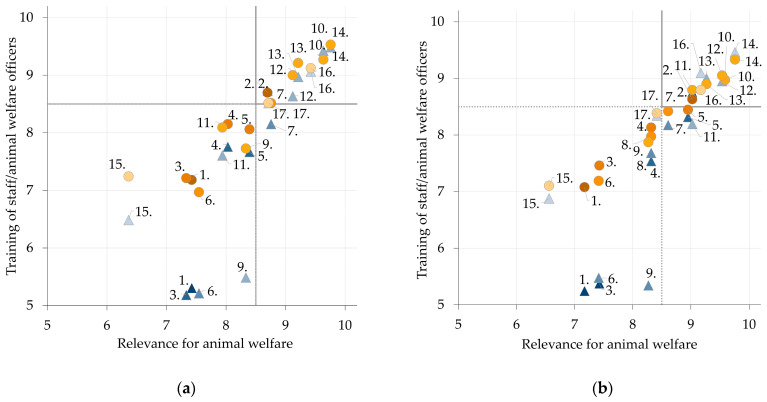
Scatterplots showing the average scores for all experts on action points in the topic areas ‘Slaughter CATTLE’ (**a**) and ‘Slaughter PIG’ (**b**) scored in the second round of the Delphi survey on animal welfare relevance and training potential of processing steps along the transport and slaughter chain of cattle and pigs. Blue triangles represent the relationship between rating of action points on ‘Relevance for animal welfare’ (*X*-axis) and ‘Training of staff’ (*Y*-axis). Orange circles represent the relationship between rating of action points on ‘Relevance for animal welfare’ (*X*-axis) and ‘Training of animal welfare officers (*Y*-axis). Different shadings of triangles and circles refer to the categorisation of the action point of the respective process step (dark to bright = process steps 1 to 5). Numbers relate to individual action points (see Supplementary File Table S1). High scores (>8.5) relate to a high relevance for animal welfare aspects and a high possibility to improve animal welfare through training. Further details can be found in Figure A2.

In total, 92/183 items (50.3%) received scores of ≥8.5. All items are presented on scatterplots (Figure 3 and Figure 4). No item received a median score rating below 5, meaning each one is at least a moderately relevant aspect of animal welfare and has a moderate possibility of improvement through targeted training. In general, items concerning constructional aspects received low ratings (Transport: action points 4., 7., 12., 15.; Slaughter: action points 1., 3., 6., 9.). In the topic area ‘Transport’ (Figure 3, see Supplementary File Table S1), the highest rated action points for both animal species included ‘Assessment of fitness for transport’ and ‘Animal welfare at unloading at abattoir’ over all three questions. For the topic area ‘Slaughter’, the action points ‘Handling at stunning’, ‘Checks on stunning’ and ‘Handling at exsanguination’ belonged to the highest rated for cattle and pigs (Figure 4, see Supplementary File Table S1).

There were no major differences between the average scores of the first and second assessment rounds for all three questions in all four topic areas.

### 3.3. Consensus Measurement

**Figure 5:**
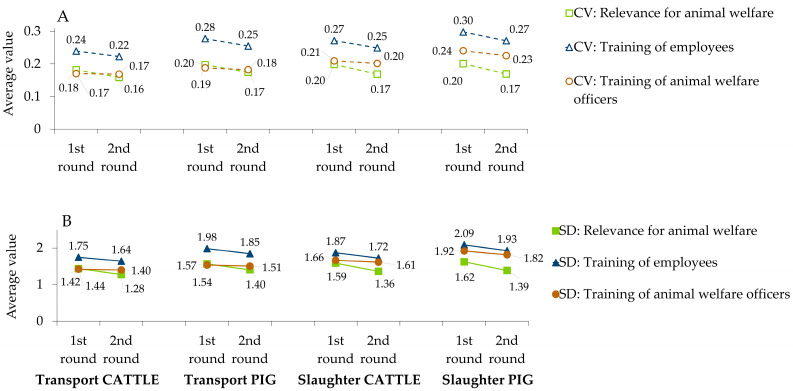
Average coefficients of variation (CV, panel **A**) and standard deviations (SD, panel **B**) calculated for the three questions (green square: ‘1. How relevant is the named action point for animal welfare aspects?’; blue triangle: ‘2. To what extent can a targeted training of staff involved in this action point improve animal welfare?’; brown circle: ‘3. To what extent can a targeted training of animal welfare officers in this action point improve animal welfare?’) and compared between the first and second round in the Delphi survey on animal welfare relevance and training potential of processing steps along the transport and slaughter chain for cattle and pigs. Reduction in CV and SD relate to an increased consensus among experts on the rating of items.

Action points were rated on 2 to 3 questions, resulting in 183 rated items. To determine the experts’ consensus on a specific rating, the SD and CV for each item were considered. The average SD and CV were calculated for each question within each topic area and compared between both rounds (Figure 5). Between the first and the second round, the average SD and CV values decreased.

In total, 92 out of the 183 items (50.3%) received high scores of ≥8.5. Their individual SD ranged between 1.42 and 2.09 in the first round, and between 1.28 and 1.93 in the second round. Of these 92 items, 85 individual SD were below the respective average SD (see Table A1, Table A2, Table A3 and Table A4); exceptions were only found for items within question number 3 (‘Training of animal welfare officers’).

## 4. Discussion

The presented modified Delphi survey assessed experts’ opinions on the importance of animal welfare aspects during transport and in the abattoir for cattle and pigs, and to what extent online training of staff and animal welfare officers may improve animal welfare. 

Relevant action points for animal welfare measures during transport and slaughter were identified based on two systematic literature reviews for cattle and pigs [15,16]. In the Delphi survey, the participating experts rated no action point below a median of 5, equalling an at least ‘moderately relevant aspect of animal welfare’, respectively ‘moderate potential of improvement’ (Figure 3 and Figure 4). The highest rated action points in the topic area ‘Transport’ for both cattle and pigs concerned assessment of fitness for transport and unloading at the abattoir. A Danish study found that 48% of drivers who transported dairy cattle could not give the right answers to two specific questions regarding the current legislation, and 35% reported to be frequently or always in doubt when assessing fitness for transport of dairy cows [19]. This report validates the experts’ assessment in the current study that ‘fitness for transport’ is a highly relevant action point for animal welfare. In the topic area ‘Slaughter’, experts rated that handling and checks on stunning, and exsanguination are highly relevant for animal welfare. Checking the sufficient stunning of an animal before exsanguination has previously been described as an extremely important aspect of animal welfare [20,21]. Correct technical execution and knowledge of the animal’s expected responses to stunning procedures are essential for adequate animal welfare-compliant stunning. All highest-rated action points include crucial steps during transport and slaughter of cattle and pigs with a focus on the recognition of significant signs of animal suffering (‘Lameness’ during ‘Unloading at abattoir’, or ‘Absence of rhythmic breathing’ at ‘Checks on stunning’). These aspects have been well documented in previous studies and are incorporated in EU legislation [15,16].

Ethically appropriate treatment of animals during transport and slaughter is part of a current public debate and governed by Regulation (EC) No 1/2005 on the protection of animals during transport and related operations [4] and Regulation (EC) No 1099/2009 on the protection of animals at the time of killing [3] in the European Union (EU). Insufficiencies of animal welfare in the everyday transportation and slaughter of cattle and pigs are well-known and under investigation for improvement [22]. Numerous options have been suggested to improve animal welfare aspects, including reduction in transportation times, optimising temperature conditions in transportation trucks, establishing standard operating procedures at abattoirs, and applying an adequate design of the restraining and stunning equipment [8,9,10,11,23]. Additionally, staff training has been named as an essential aspect to improve animal welfare by the European Food Safety Authority (EFSA) Panel on Animal Health and Welfare [8,9,10,11] and in further reports [19,24,25,26,27,28,29,30,31,32,33,34,35,36]. In livestock production, handling of animals is regarded as one of the most influential factors affecting animal welfare [27,28,37]. One report has shown that training staff on behavioural principles of handling pigs can greatly reduce the use of electric prods [24]. Another study reported that abattoir staff had better knowledge and higher confidence to improve animal welfare following training [25]. However, attitudes towards importance of animal welfare did not change following training [25]. Several authors describe education and training of staff as an effective means to minimise stress in cattle on farms and before slaughter [26,38,39,40]. Gaining basic and consistent knowledge regarding an animal’s behavioural patterns and its welfare requirements is regarded as a fundamental part of training [27]. Experts in the current study have rated recognition of behavioural patterns such as fear and stress during loading onto a trailer, unloading at abattoir and stunning as highly relevant action points (rating > 8.5) for animal welfare. In agreement with previous studies, they have additionally been rated as having a high potential for improvement through training of staff (Table A1, Table A2, Table A3 and Table A4).

Effective training materials need to be designed and implemented by qualified individuals and presented in the appropriate language and at the suitable educational level [41]. Online training has been reported as an effective tool to teach animal welfare [42]. Distinguished graphical presentations can support knowledge acquisition, especially for those with little prior knowledge or education, and the design of a modular training program allows for learners to train at their own pace [43,44]. The action points identified as highly relevant for animal welfare serve as the basis for the development of online training modules for staff and animal welfare officers at transport and in the abattoir. An additional important factor regarding staff’s attitude towards animal welfare are working conditions and management style [27]. Future studies may investigate on how exactly these factors relate to staff’s behaviour, the subsequent impact on animal welfare and whether (online) training is a suitable tool for improvement.

### 4.1. Validity and Expert Subgroups

To assess the validity of the modified Delphi survey, response rate (47.1% in the first round), dropout rate between the first and second round (16.3%) and number of participants (49 experts) were considered. Response rates for Delphi surveys tend to vary; however, a participation rate of at least 70% is recommended [45]. The dropout rate is subject to fluctuations. Keeney et al. [45] documented studies with each 28% and 40% dropout rates. They also reported that typical sample sizes include 10 to 100 participants [45], whereas Hasson et al. (2000) mentioned 15 to 60 individuals for a sufficient analysis [46]. For this Delphi survey, it was concluded that the low dropout rate and the resulting reasonable sample size were sufficient to achieve the survey’s objectives despite the relatively low response rate. Further considerations include the complex nature of the survey tool, an MS Excel^©^-based workbook with six spreadsheets, and the worldwide pandemic situation around COVID-19 in the year of 2021.

Between the three expert subgroups, half of the participants were experts based in research institutions focusing on either animal welfare or food science. The fact that no significant differences in average scores were seen between the three subgroups indicate that the survey results were not detectably biased by the larger cohort of experts from the research domain. 

### 4.2. Measuring Consensus

Delphi surveys are used to reach a consensus for an unknown issue [17]. The individual research group predetermines the level of consensus, which is often measured in percent [45]. Typical rates which are defined as consensus range from 51% to 80% [46]. As the presented scale includes eleven individual numbers, consensus was not calculated for each scale point, but it was decided to focus on the average SD and CV for each topic area (Figure 5). 

As SD is commonly used as a measurement of consensus, it was chosen to include it in the interpretation of the results despite the skewed distribution of the data. A numerically and relatively small SD of scores reflects a high consensus among experts on a specific item. The exact measurement of SD as a value for consensus is undefined. West and Cannon [47] used a value of 1.64 (on a 4-point Likert-type scale) and Christie and Barela [48] suggested a cut-off of 1.5. Others have used SD as an indicator to estimate the trend of consensus between rounds [49,50,51], with a decreasing SD equalling greater consensus between experts. In the current survey, the average SDs were calculated for each topic area regarding the three questions mentioned above. These averages decreased between rounds one and two, showing an increased consensus for all topic areas (Figure 5). Out of the 92 items scored ≥ 8.5, the SD of 85 items were below the respective average, showing that the experts reached consensus especially on the particular importance of the highest-rated items. This consensus therefore enhances the relevance of these items for animal welfare and the potential of improvement through training.

An additional indicator to define consensus is the CV. English and Kernan (1976) defined a CV of <0.5 as a ‘good degree of consensus’, which does not require an additional round. Another study confirmed a consistent decrease in the CV to indicate an increase in consensus [52]. In the first round, the average CV values were already below 0.5 and became even smaller in the second round (Figure 5). The obtained differences between both rounds were between 0.00 and 0.03. These minor differences are indicative of increased consensus among the experts after two rounds and deem an additional round unnecessary.

When comparing the methods of measuring consensus (SD vs. CV), it should be noted that the survey’s CV values suggested a particularly high consensus between experts, whereas the SD values were not necessarily within the cut-off limits suggested by previous studies [47,48,53,54]. This is due to the comparatively large rating scale of eleven individual numbers (0 to 10), whose means will have higher SD and smaller CV than a scale with fewer scale points. Due to this dependency, assessing consensus does not have absolute criteria, and must be performed with caution.

Another survey method to reach consensus is the Nominal Group Technique (NGT) [55,56]. Here, participants interact face-to-face. In the first step, participants document their opinions or ideas independently. In the group phase, participants take turns in sharing items from their list before engaging in a discussion. A consensus can be reached much faster than using the Delphi approach, but the number of participants is limited [55,56]. The use of NGT in this study could have contributed to a clearer distinction between the rating of action points, as the participating experts could have made greater differentiations regarding relevance for animal welfare in an open discussion. However, as this particular area of expertise is small and the participating experts come from different places, finding a suitable time slot could have resulted in a low number of participants.

### 4.3. Limitations

The results of relevance for animal welfare and trainability were generally rated as equally high. This could be a bias of the data collection, as the participants of the Delphi survey received a MS Excel^©^-based workbook in which the trainability was right next to the relevance for animal welfare, and high scores on the relevance score (asked first) could have influenced the trainability score (Figure 1, Supplementary File Table S1). The project team has chosen to compromise here, as collecting scores on different worksheets would have resulted in twelve individual spreadsheets to be completed instead of four. However, there is a reasonable connection between an item’s necessity for improved animal welfare and the possibility to train this item (excluding constructional conditions). 

The convenience sample of experts included in the approach could have biased the results; however, a systematic distortion was not visible beyond having received rather high average scores on all action points. 

## 5. Conclusions

The expert elicitation using a Delphi approach was well suited to identifying highly relevant action points where animal welfare can be improved through staff training at transport and slaughter of cattle and pigs. In the second survey round, the variability in experts’ responses was further reduced towards consensus. This study highlights the high level of consensus among experts from different backgrounds, including research institutions, meat industry and meat inspection/animal welfare, on the need for training tools focusing on specific activities to improve animal welfare during transport and slaughter. Topics of particular relevance to animal welfare included assessment of fitness for transport, animal welfare at unloading at the abattoir, handling at stunning, checks on stunning and handling at exsanguination. The development of online training modules for transport companies as well as for staff and animal welfare officers at abattoirs focuses on the highest-rated action points. 

## Figures and Tables

**Table 1:** Overview of topic areas and process steps considered in the expert elicitation (modified Delphi) approach to identify action points considered most relevant for the training platform.

	Topic Areas	Topic Areas
	‘Transport CATTLE/PIG’	‘Slaughter CATTLE/PIG’
Process steps	1. Route planning/time management	1. Moving to lairage pen
	2. Moving to loading area	2. Lairage pen
	3. Loading	3. Moving to stunning area
	4. Transportation	4. Stunning
	5. Unloading at the abattoir	5. Exsanguination

**Table 2:** Categories of experts chosen for the Delphi survey on animal welfare relevance and training potential of processing steps along the transport and slaughter chain of cattle and pigs.

Category	Field of Expertise
Experts based in research institutions	Professors, university lecturers,
	scientists from different research institutions
Experts based in the industry	Food business operators, quality assurance managers, transporters
Experts based in meat inspection/	Animal welfare officers, official veterinarians in meat inspection/animal welfare
animal welfare	

**Table 3:** Selected comments from the second round of the Delphi survey on animal welfare relevance and training potential of processing steps along the transport and slaughter chain for cattle and pigs.

Topic Area	Process Step:	Question	Median Value	Expert’s Score in the 1st Round	Expert’s Reassessment in the 2nd Round
	Action Point				
Transport	Transportation:	2 (Training of staff)	8	3	3
CATTLE	Driving				
**Comment:** Targeted training cannot influence the traffic situation, which is responsible for travel stops, vibrations and transport duration.					
Transport PIG	Unloading at abattoir: Animal behaviour	2 (Training of staff)	8.5	5	5
**Comment:** Training may improve one’s knowledge on the topic and possibly raise awareness among staff. However, my low score refers to the doubt that this will lead to changes in everyday life in the longer term.					
Slaughter	Moving to lairage pen: Constructional	1 (Relevance for	7	4	6
CATTLE		animal welfare)			
**Comment:** As Temple Grandin pointed out, constructional conditions have an influence on animal welfare. However, I consider this to be less relevant in relation to handling.					
Slaughter PIG	Lairage pen:	3 (Training of animal welfare officers)	8	3	5
	Handling				
**Comment:** Alignment with median, but animal welfare officers will not be able to strongly influence the staff’s behaviour through their own behaviour, they would have to check the staff in this aspect. I do not know if such a thing is feasible.					

**Figure A1:**
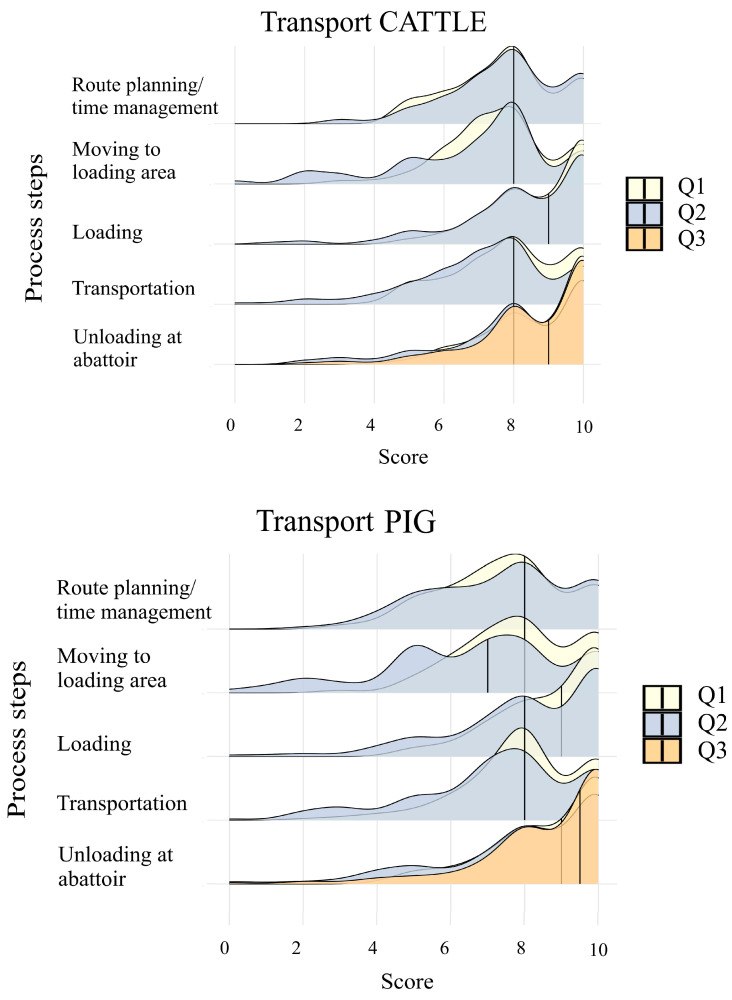
Ridgeline-plots for the distributions of answers for the topic areas ‘Transport CATTLE’ (top panel) and ‘Transport PIG’ (bottom panel) scored in the second round of the Delphi survey on animal welfare relevance and training potential of processing steps along the transport and slaughter chain of cattle and pigs. Q1, Question 1: ‘How relevant is the named action point for animal welfare aspects?’; Q2, Question 2: ‘To what extent can a targeted training of staff involved in this action point improve animal welfare?’; Q3, Question 3: ‘To what extent can a targeted training of animal welfare officers in this action point improve animal welfare?’.

**Figure A2:**
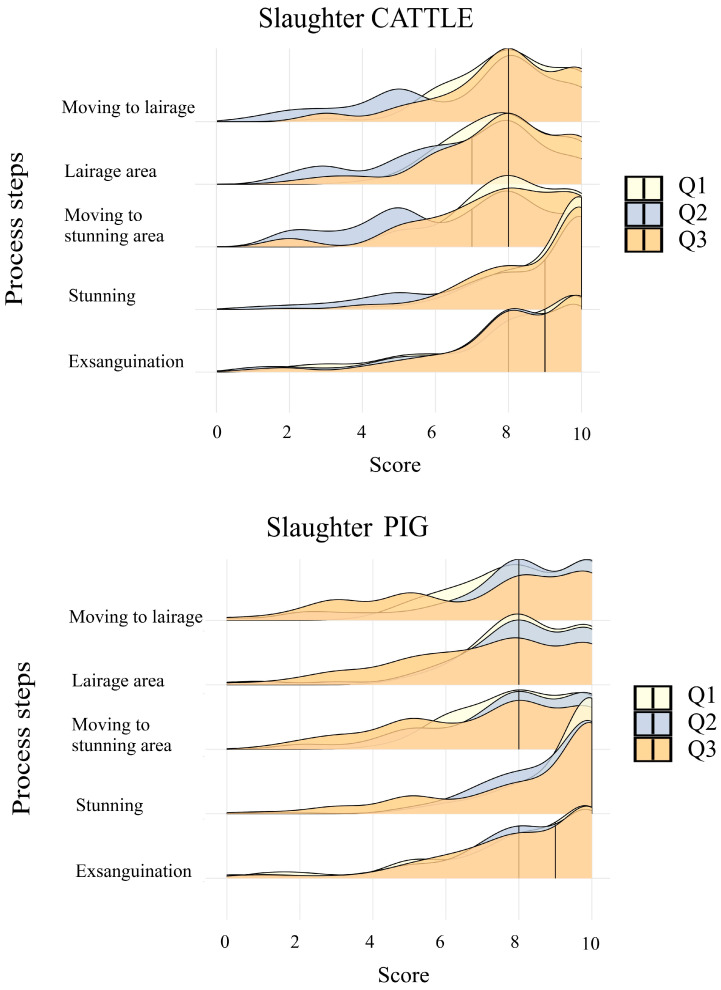
Ridgeline-plots for the distributions of answers for the topic areas ‘Slaughter CATTLE’ and ‘Slaughter PIG’ scored in the second round of the Delphi survey on animal welfare relevance and training potential of processing steps along the transport and slaughter chain of cattle and pigs. Q1, Question 1: ‘How relevant is the named action point for animal welfare aspects?’; Q2, Question 2: ‘To what extent can a targeted training of staff involved in this action point improve animal welfare?’; Q3, Question 3: ‘To what extent can a targeted training of animal welfare officers in this action point improve animal welfare?’.

## Data Availability

The data presented in this study are available on request from the corresponding author. The data are not publicly available due to privacy reasons.

## Supplementary Materials

The following supporting information can be downloaded at: https://www.mdpi.com/article/10.3390/ani13243859/s1.

## Appendix A

### Appendix A.2. Standard Deviations of Topic Areas

**Table A1:** Action points with the highest mean scores (≥8.5) in the topic area “Transport CATTLE” of the Delphi survey on animal welfare relevance and training potential of processing steps along the transport and slaughter chain of cattle and pigs.

Transport CATTLE				
Process step: Action Point (Number) with a Score of ≥8.5 for Q1	First Round:		Second Round:	
	Q2 ≥ 8.5		Q2 ≥ 8.5	
	Q3 ≥ 8.5		Q3 ≥ 8.5	
	Mean ± SD	CV	Mean ± SD	CV
Loading: Assessment for fitness of transport (6.)	Q2 (9.51 ± 0.87)	0.09	Q2 (9.51 ± 0.87)	0.09
Loading: Handling and Herding (8.)	Q2 (8.68 ± 1.20)	0.14	Q2 (8.78 ± 1.11)	0.13
Loading: Animal behaviour (9.)	Q2 (8.70 ± 1.51)	0.17	Q2 (8.89 ± 1.24)	0.14
Unloading at abattoir: Handling (16.)	Q3 (8.55 ± 1.73) *	0.20 *	Q3 (8.55 ± 1.73) *	0.20 *
Unloading at abattoir: Animal behaviour (17.)	Q2 (8.50 ± 1.67)	0.20	Q2 (8.58 ± 1.56)	0.18
	Q3 (8.71 ± 1.42)	0.16	Q3 (8.71 ± 1.42)	0.16
Unloading at abattoir: Animal welfare/	Q2 (9.12 ± 1.24)	0.13	Q2 (9.19 ± 1.24)	0.13
animal health (18.)	Q3 (9.45 ± 0.85)	0.09	Q3 (9.45 ± 0.85)	0.09
Unloading at abattoir: Animal welfare-related	Q2 (8.94 ± 1.31)	0.14	Q2 (8.94 ± 1.31)	0.15
handling (19.)	Q3 (9.36 ± 0.92)	0.10	Q3 (9.36 ± 0.92)	0.10

Q1, Question 1: “How relevant is the named action point for animal welfare aspects?”; Q2, Question 2: “To what extent can a targeted training of staff involved in this action point improve animal welfare?”; Q3, Question 3: “To what extent can a targeted training of animal welfare officers in this action point improve animal welfare?”; SD, standard deviation; CV, coefficient of variation; * SDs and CVs above average (see Figure 5) are marked with an asterisk.

**Table A2:** Action points with the highest mean scores (≥8.5) in the topic area “Transport PIG” of the Delphi survey on animal welfare relevance and training potential of processing steps along the transport and slaughter chain of cattle and pigs.

Transport PIG				
Process step: Action Point (Number) with a Score of ≥8.5 for Q1	First Round:		Second Round:	
	Q2 ≥ 8.5		Q2 ≥ 8.5	
	Q3 ≥ 8.5		Q3 ≥ 8.5	
	Mean ± SD	CV	Mean ± SD	CV
Loading: Assessment for fitness of transport (6.)	Q2 (9.38 ± 1.17)	0.12	Q2 (8.67 ± 1.16)	0.12
Loading: Handling and Herding (8.)	Q2 (8.81 ± 1.31)	0.15	Q2 (9.38 ± 1.17)	0.13
Unloading at abattoir: Handling (16.)	Q3 (8.59 ± 1.85) *	0.21 *	Q3 (8.62 ± 1.83) *	0.21 *
Unloading at abattoir: Animal behaviour (17.)	Q3 (8.72 ± 1.47)	0.17	Q3 (8.77 ± 1.42)	0.16
Unloading at abattoir: Animal welfare/	Q2 (8.98 ± 1.55)	0.17	Q2 (8.98 ± 1.55)	0.17
animal health (18.)	Q3 (9.41 ± 0.88)	0.09	Q3 (9.41 ± 0.88)	0.09
Unloading at abattoir: Animal welfare-related	Q2 (8.92 ± 1.44)	0.16	Q2 (8.92 ± 1.44)	0.16
handling (19.)	Q3 (9.41 ± 0.85)	0.09	Q3 (9.41 ± 0.85)	0.09

Q1, Question 1: “How relevant is the named action point for animal welfare aspects?”; Q2, Question 2: “To what extent can a targeted training of staff involved in this action point improve animal welfare?”; Q3, Question 3: “To what extent can a targeted training of animal welfare officers in this action point improve animal welfare?”; SD, standard deviation; CV, coefficient of variation; * SDs and CVs above average (see Figure 5) are marked with an asterisk.

**Table A3:** Action points with the highest mean scores (≥8.5) in the topic area “Slaughter CATTLE” of the Delphi survey on animal welfare relevance and training potential of processing steps along the transport and slaughter chain of cattle and pigs.

Slaughter CATTLE				
Process step: Action Point (Number) with a Score of ≥8.5 for Q1	First Round:		Second Round:	
	Q2 ≥ 8.5		Q2 ≥ 8.5	
	Q3 ≥ 8.5		Q3 ≥ 8.5	
	Mean ± SD	CV	Mean ± SD	CV
2. Herding to holding pen: Handling	Q2 (8.67 ± 1.11)	0.13	Q2 (8.73 ± 1.01)	0.12
	Q3 (8.67 ± 1.27)	0.15	Q3 (8.70 ± 1.24)	0.14
7. Herding to stunning area: Handling	Q3 (8.52 ± 1.28)	0.15	Q3 (8.52 ± 1.28)	0.15
10. Stunning: Handling	Q2 (9.42 ± 1.06)	0.11	Q2 (9.42 ± 1.06)	0.11
	Q3 (9.27 ± 1.18)	0.13	Q3 (9.27 ± 1.18)	0.13
12. Stunning: Animal behaviour	Q2 (8.64 ± 1.41)	0.16	Q2 (8.64 ± 1.41)	0.16
	Q3 (9.00 ± 1.12)	0.12	Q3 (9.00 ± 1.12)	0.12
13. Stunning: Repeated stunning	Q2 (8.91 ± 1.79)	0.20	Q2 (8.97 ± 1.72)	0.19
	Q3 (9.21 ± 1.41)	0.15	Q3 (9.21 ± 1.41)	0.15
14. Stunning: Checks on stunning	Q2 (9.49 ± 1.06)	0.11	Q2 (9.49 ± 1.06)	0.11
	Q3 (9.53 ± 1.08)	0.11	Q3 (9.53 ± 1.08)	0.11
16. Exsanguination: Handling	Q2 (8.91 ± 1.55)	0.17	Q2 (9.06 ± 1.37)	0.15
	Q3 (8.91 ± 1.51)	0.17	Q3 (9.12 ± 1.22)	0.13
17. Exsanguination: Checks on exsanguination	Q2 (8.52 ± 1.40)	0.16	Q2 (8.52 ± 1.40)	0.16
	Q3 (8.52 ± 1.44)	0.17	Q3 (8.52 ± 1.44)	0.17

Q1, Question 1: “How relevant is the named action point for animal welfare aspects?”; Q2, Question 2: “To what extent can a targeted training of staff involved in this action point improve animal welfare?”; Q3, Question 3: “To what extent can a targeted training of animal welfare officers in this action point improve animal welfare?”; SD, standard deviation; CV, coefficient of variation.

**Table A4:** Action points with the highest mean scores (≥8.5) in the topic area “Slaughter PIG” of the Delphi survey on animal welfare relevance and training potential of processing steps along the transport and slaughter chain of cattle and pigs.

Slaughter PIG				
Process step: Action Point (Number) with a Score of ≥8.5 for Q1	First Round:		Second Round:	
	Q2 ≥ 8.5		Q2 ≥ 8.5	
	Q3 ≥ 8.5		Q3 ≥ 8.5	
	Mean ± SD	CV	Mean ± SD	CV
2. Herding to holding pen: Handling	Q2 (8.68 ± 1.62)	0.19	Q2 (8.76 ± 1.51)	0.17
	Q3 (8.53 ± 1.93) *	0.23	Q3 (8.63 ± 1.72)	0.20
10. Stunning: Handling	Q2 (8.98 ± 1.68)	0.19	Q2 (9.02 ± 1.62)	0.18
	Q3 (8.92 ± 1.77)	0.20	Q3 (8.97 ± 1.71)	0.19
11. Stunning: Stunning methods	Q3 (8.74 ± 1.53)	0.18	Q3 (8.80 ± 1.44)	0.16
12. Stunning: Animal behaviour	Q2 (8.83 ± 1.61)	0.18	Q2 (8.95 ± 1.51)	0.17
	Q3 (8.97 ± 1.35)	0.15	Q3 (9.05 ± 1.30)	0.14
13. Stunning: Repeated stunning	Q2 (9.00 ± 1.86)	0.21	Q2 (9.00 ± 1.86)	0.21
	Q3 (8.90 ± 1.37)	0.15	Q3 (8.90 ± 1.37)	0.15
14. Stunning: Checks on stunning	Q2 (9.42 ± 1.18)	0.13	Q2 (9.46 ± 1.16)	0.12
	Q3 (9.28 ± 1.12)	0.12	Q3 (9.33 ± 1.11)	0.11
16. Exsanguination: Handling	Q2 (8.95 ± 1.41)	0.16	Q2 (9.10 ± 1.30)	0.14
	Q3 (8.74 ± 1.41)	0.16	Q3 (8.80 ± 1.34)	0.15

Q1, Question 1: “How relevant is the named action point for animal welfare aspects?”; Q2, Question 2: “To what extent can a targeted training of staff involved in this action point improve animal welfare?”; Q3, Question 3: “To what extent can a targeted training of animal welfare officers in this action point improve animal welfare?”; SD, standard deviation; CV, coefficient of variation; * SDs and CVs above average (see Figure 5) are marked with an asterisk.
